# Representation of ethnic and racial minority groups in European vaccine trials: a quantitative analysis of clinical trials registries

**DOI:** 10.1136/bmjph-2023-000042

**Published:** 2023-10-18

**Authors:** Natasha F Puttick, Samantha Vanderslott, Rachel Tanner

**Affiliations:** 1 Keble College, University of Oxford, Oxford, UK; 2 Oxford Vaccine Group, Department of Paediatrics, University of Oxford, Oxford, UK; 3 Centre for Clinical Vaccinology and Tropical Medicine, NIHR Oxford Biomedical Research Centre, Oxford, UK; 4 Department of Biology, University of Oxford, Oxford, UK

**Keywords:** Public Health, Disease Transmission, Infectious, methods

## Abstract

**Objectives:**

The representation of ethnic minority groups in European vaccine trials is an important and hitherto unaddressed gap in the literature. The objectives of this study were to determine the proportion of European vaccine trials that report data on the ethnic demographics of participants, to evaluate the distribution of ethnic minority groups among trial participants (where reported), and ascertain whether this is representative of the wider population of the country.

**Design:**

We evaluated the representation of ethnic/racial minority groups in clinical research, conducting a quantitative analysis of clinical trials registry data from completed vaccine trials in Europe that commenced between 1 January 2010 and 31 December 2020.

**Data sources:**

Data were collected from four major clinical trial databases: ClinicalTrials.gov, the European Union Clinical Trials Register (EUCTR), the International Standard Randomised Controlled Trial Number (ISRCTN) and the International Clinical Trials Registry Platform (ICTRP).

**Results:**

A majority of clinical trials failed to either record or report the race/ethnicity of their volunteers on the clinical trials registry databases. Reported participants in UK vaccine trials were not representative of the ethnic demographics of the wider population. Unavailability of population-level ethnicity data for many European countries was a significant barrier to determining the wider applicability of these findings.

**Conclusions:**

Under-representation of ethnic minority groups in vaccine trials may have implications for the effectiveness of routine vaccinations, threatening the principles of justice and equity that are embedded in national medical research guidelines. Unavailability of population-level ethnicity data exacerbates the prevailing lack of understanding of the extent of this issue, despite literature indicating cause for concern.

**WHAT IS ALREADY KNOWN ON THIS TOPIC**

- Prior to this study, the representation of ethnic minority groups in clinical trials investigating a range of disease and health conditions has been considered including for diabetes, progressive diseases and health technology research.
- However, there is a dearth of previous literature assessing the inclusion of racial/ethnic minority volunteers in clinical vac­cine trials with the exception of a recent report from the USA.

**WHAT THIS STUDY ADDS**

- We present the first study to collate and analyse participant data from clinical trials registries for vaccine trials conducted in Europe.
- Our findings indicate the under-representation of ethnic minority groups in UK vac­cine trials and the failure in most cases to record or report the ethnicity of European vaccine trial partici­pants in clinical trials databases.

**HOW THIS STUDY MIGHT AFFECT RESEARCH, PRACTICE OR POLICY**

- Under-representation of ethnic minority groups in vaccine trials may have practical implications in terms of erroneously generalising the safety or efficacy of routine vaccinations, and threatens the principles of justice and equity embedded in national guidelines on medical research.
- Systemic and con­textual barriers to inclusion must be addressed, with advocation for a change to the framework of data collection for clinical trials registries.

## Introduction

 The value of well-constructed medical research to society is difficult to overstate. Medical research can provide crucial information on disease trends, risk factors, public health interventions, care outcomes and treatment applications.^1^ Phased clinical trials in an appropriately compensated subset of human volunteers are essential to assess the safety and efficacy of medical interventions such as vaccines and therapeutics. However, the value of such trials depends on the generalisability of findings to the target population; a volunteer cohort in a clinical trial that is broadly unrepresentative of the population to which the intervention will be applied yields limited predictive value.

It is important to first set out that the acknowledgement of race as a variable within research can be controversial, with some arguing it represents a socially constructed and misleading understanding of biological differences.^2^ Therefore, certain representations of race have potential to not only harm human health but risk reinforcing the narrative of essential between-group differences.^3^ Nonetheless, the importance of representation in clinical research goes beyond ensuring generalisability of findings: it also concerns justice and equity. Lack of inclusion of any group goes against the principles of justice embedded in national and international research guidelines. For example, the Belmont report of 1979 included the notion of justice as permittance to the benefits of research, having direct implications for groups that are understudied whether unintentionally or systemically.^4^ Even if inclusion is not recognised as an issue of medical significance, it remains one of ethical consequence.

It is also important to elucidate the terms ‘race’ and ‘ethnicity’ which appear in contested variations throughout the literature. The term ‘race’ has been subject to debate but may be defined as ‘a biological concept which categorises humanity by means of sets of phenotypical features that appear to distinguish between varieties of people and are passed on between generations’.^5^ There have been calls to abandon the concept of ‘race’ in the context of medical research arguing it represents a painful and divisive relic,^6^ though this may be too simplistic a solution.^7^ Nonetheless, race remains a flawed concept: socially constructed with poorly defined genetic markers applied inconsistently. Thus, the terms ‘ethnicity’/‘ethnic minority’/‘ethnic group’ will be preferentially used henceforth. Ethnicity can be defined as a ‘social group with a shared history, lineage, heritage, sense of identity, cultural roots, and territorial identity’.^8^ Importantly, the term ‘ethnic minority groups’ is used to refer only to a collective of individualised groups, not to imply any aspect of homogeneity between these populations.

The nature of prophylactic vaccines means they are usually designed to be administered homogeneously across entire healthy populations or age groups (as opposed to therapeutics targeting subgroups of patients), necessitating accurate representation of the target population during trials. Representing all demographic groups is central to reducing the risk of unexpected adverse events or lower than expected immunogenicity and/or efficacy during vaccine roll-out. When such events occur, they may lessen public trust in vaccines.^9^ Furthermore, a vaccine designed for global use proving less effective in subsets of the population compromises overall effectiveness; this is particularly pertinent in an age of globalisation and increased risk of pandemics. Comparative studies of outcomes between different racial/ethnic groups have highlighted the potential relevance of representation in vaccine trials. Ethnicity has been associated with significant variation in immunogenicity and/or efficacy of vaccines against influenza A,^10^ hepatitis B^11 12^ and SARS-CoV-2^13^ among others.

Given the dearth of research on the participation of ethnic minority groups in European vaccine trials, we performed a novel analysis of reported data over a 10-year period to further understanding of participation rates and the extent of their representation in the wider population. This study set out to answer three key questions: (1) what proportion of European vaccine trials report data on the ethnic demographics of participants in clinical trials registries?; (2) in trials that do report ethnic demographics of participants in registries, what is the distribution of ethnic minority groups? and (3) is the distribution of ethnic minority groups in European vaccine trials representative of the wider population of the country in which they are conducted?

## Methods

### Information sources and search terms

Data were collected from four major clinical trial databases: ClinicalTrials.gov, the European Union Clinical Trials Register (EUCTR), the International Standard Randomised Controlled Trial Number (ISRCTN) registry and the International Clinical Trials Registry Platform (ICTRP). The search terms used were ‘Vaccine’, ‘Vaccination’, ‘Immunisation’ and ‘Inoculation’ in British and American English permutations. Some of the registries automatically searched for related terms; if this feature was used, the map of related terms of that registry was assessed to ensure it contained all relevant terms. No geographical terms or filters were included as reporting of trial location differed between registries, and continuity of searches was prioritised due to the likelihood of a trial being registered on multiple databases.

### Data collection, aggregation and cleaning

Data were collected from vaccine trials fulfilling the criteria defined in table 1, filtering for trials that commenced between 1 January 2010 and 31 December 2020, and for those with reported results. Each hit from each registry was manually examined using the exclusion criteria. The first factor examined was whether the trial related to a prophylactic vaccine; trials related to therapeutic vaccines were excluded on the basis that (1) they are not intended for deployment across the general population, rather in a subset of patients with a particular disease and (2) the study populations are biased by the demographics of patients with that disease. Trial location was then determined and those including populations outside of Europe were excluded. Trials were also excluded if they were suspended or prematurely terminated, if they were only recorded in short form, or if it was not evident what region the participants were recruited from. If a trial passed this first round of parsing, details were extracted and recorded using Microsoft Excel, including: search term, trial registry, recruitment start data, nature of targeted disease, number of participants, digital object identifier, study design and whether ethnicity of participants was recorded on the registry. Trials reporting only estimates or aims for numbers of participants were excluded from the final analysis. Data were not collected if only reporting the percentage of Hispanic versus non-Hispanic participants.

**Table 1:** Criteria for vaccine trials from which data were collected

Commenced between 1 January 2010 and 31 December 2020.
Registered on a publicly accessible clinical trials registry from the WHO list of primary registries.
Recruitment of study participants from European nation(s) only. Clinical trials based in Russia were excluded on the following basis: (1) Although the majority of its population is located in Europe, approximately 77% of Russia’s landmass is in Asia; (2) Russia is a demographically unique nation with a large range of ethnicities spanning a huge landmass, thus limiting the relevance of findings to Europe more widely.
Consisting of a clinical trial in any phase from 1 to 4 relating to prophylactic vaccine development. Therapeutic vaccines were excluded on the following basis: (1) they are not intended for deployment across the general population, rather in a subset of patients with a particular disease; and (2) the study populations are biased by the demographics of patients with that disease.
Published in English.
Trial completed with results available.
Trials were excluded if they were: (1) suspended or prematurely ended; (2) only recorded in short form; or (3) it was not evident which region study participants were recruited from.

A heterogeneous range of ethnic group labels was used within clinical trials reporting. For effective data collection, the most commonly used labels were selected as defined in table 2, and the wider data allocated to these categories. After all captured trials had been manually parsed and relevant data recorded, duplicate trials were removed, the dataset manually examined for omissions or inconsistencies, and the data aggregated to form the final dataset. This included clinical trials recruiting participants from multiple European nations, although only a subset of countries was included in the final statistical analysis as described in Reporting of ethnicity data. Contingency tables were created from data for countries identified for further analysis.

**Table 2:** Ethnic group categories selected for collection of data on the representation of ethnic minority groups in vaccine trials (adapted from ‘Racial and Ethnic Categories and Definitions for NIH Diversity Programs and for Other Reporting Purposes’ described in NIH NOT-OD-15-089, April 2015).

Asian	A person having origins in any of the original peoples of the Far East, Southeast Asia, or the Indian subcontinent including, for example, Cambodia, China, India, Japan, Korea, Malaysia, Pakistan, the Philippine Islands, Thailand, and Vietnam.
American Indian or Alaska Native	A person having origins in any of the original peoples of North and South America (including Central America), and who maintains tribal affiliation or community attachment.
Native Hawaiian or Other Pacific Islander	A person having origins in any of the original peoples of Hawaii, Guam, Samoa or other Pacific Islands.
Black or African American	A person having origins in any of the black racial groups of Africa.
Middle Eastern or North African	A person having origins in any of the original peoples of the Middle East or North Africa.
White European	A person having origins in any of the original peoples of Europe.
More than one race, unknown or otherwise reported	

### Statistical analysis

Statistical analysis was conducted by using RStudio for Windows, R V.4.1.2, and Microsoft Excel for Windows 2022. Data were aggregated and cleaned in Microsoft Excel prior to importing into R. For the comparison between trial and population-level demographic data, a χ^2^ goodness-of-fit test was conducted with a significance cut-off of p<0.05.

## Results

### Reporting of ethnicity data

**Figure 1:**
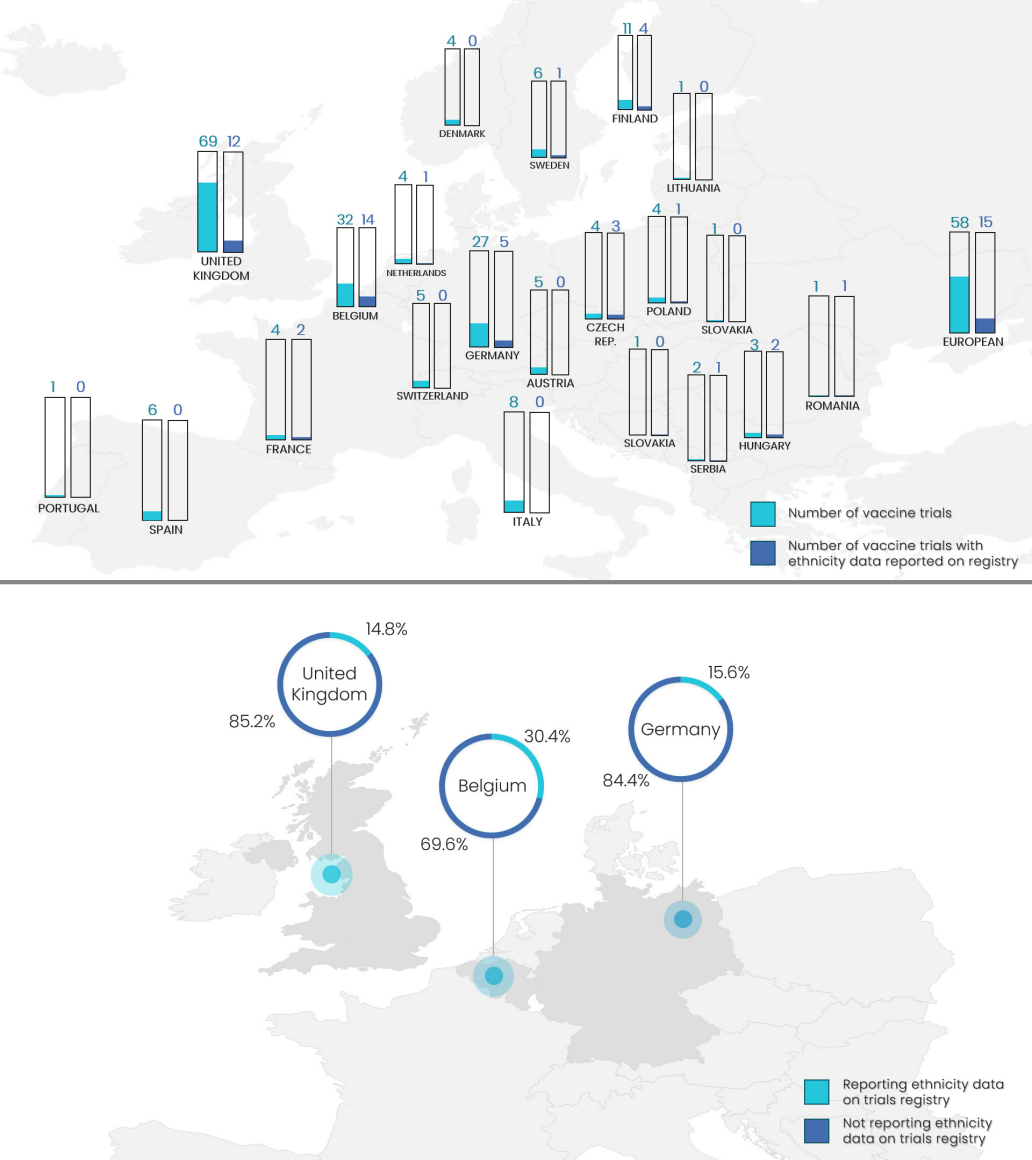
**(Top**) The number of vaccine trials included in the analysis (green) by European country and of these, the number of trials reporting ethnicity data on the registry (blue). (**Bottom**) The percentage of vaccine trials reporting ethnicity data (green) and not reporting ethnicity data (blue) on the registry for included vaccine trials conducted in Belgium, Germany and the UK.

The number of vaccine trials that commenced between 2010 and 2020 with reported data included in the analysis was variable with the highest numbers in the UK (69), Belgium (32) and Germany (27) (figure 1A). The mean proportion of these trials reporting ethnicity data in the registries across all European countries was 26%, although this may be inflated by countries such as Romania, for which only one trial was included and ethnicity data was reported, giving a value of 100% (figure 1A). While the Czech Republic ranked second highest for ethnicity data reporting, the small number of vaccine trials (four) registered during this time-frame may again have skewed results. To avoid potentially misleading findings, subsequent analysis focused on a subset of countries (Belgium, Germany and the UK) selected according to the following criteria: (1) the most common locations for clinical trials in Europe; (2) the most common study locations in the data collected and (3) the study locations that recorded more than 15 clinical trials reporting ethnicity data. Of included vaccine trials in Belgium, Germany and the UK, 14/32 (44%), 5/27 (19%) and 12/69 (17%), respectively, reported participant ethnicity data in the registry (figure 1B).

### Representation of ethnic minority groups

**Figure 2:**
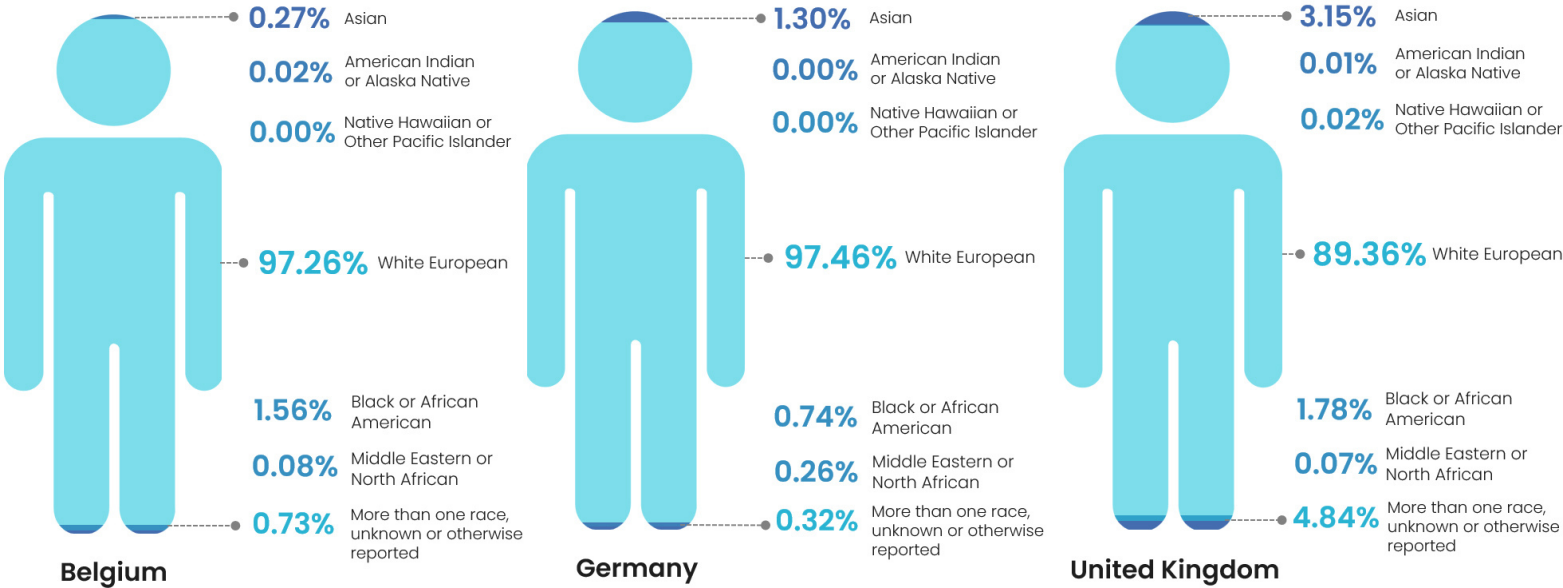
Representation of ethnic minority groups in vaccine trials that commenced between 2010 and 2020 conducted in Belgium, Germany and the UK with reported ethnicity data on the registry.

The mean demographic of aggregated data from included vaccine trials conducted in Belgium was 93.53% white European, 0.61% Asian and 45.02% black or African American, with all other categories <1%. However, these results were skewed by a single outlier study for which the ethnicity breakdown was 5% Asian, 50% black or African American and 45% white European. When this study was excluded, the mean ethnic breakdown was 97.26% white European, 0.27% Asian and 1.56% black or African American (figure 2A). Participants in trials conducted in Germany were 97.46% white European, 1.3% Asian and 0.74% black or African American participants. All other categories reported either 0 or <1% participation rates. There was no representation of mixed-race participants (figure 2B). By comparison, statistics for included vaccine trials conducted in the UK showed 89.36% white European, 3.15% Asian, 1.78% black or African American, 21.64% more than one race and 3.2% participants of unknown or otherwise reported ethnicity (figure 2C).

**Figure 3:**
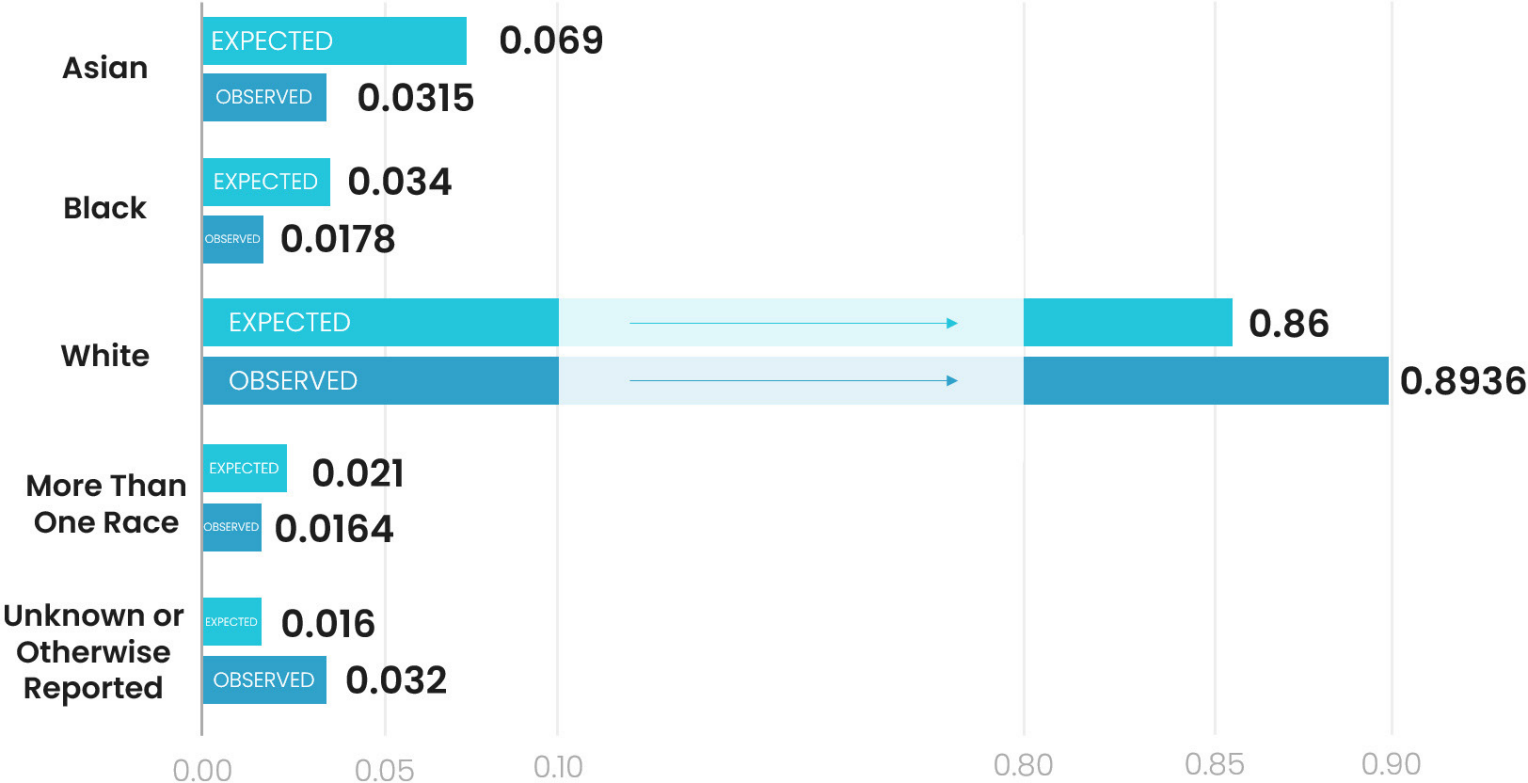
Proportion of participants across ethnic groups ‘expected’ in UK vaccine trials according to the wider population demographics (green) compared with the proportion of participants ‘observed’ in included vaccine trials conducted in the UK with reported data on the registry (blue).

Using aggregated collected data on included UK vaccine trials and data from the UK Annual Population Survey, there was a significant difference in the distributions of participants across ethnic groups between reported UK vaccine trial participants and the wider UK population (χ^2^=156.51, p<0.0001). White European participants were over-represented in vaccine trials compared with UK ethnic demographic data (0.89 observed compared with 0.86 expected, an increase of 3.91%), while black or African American and Asian racial/ethnic groups were under-represented (figure 3). Asian participants were particularly under-represented (0.069 expected compared with 0.0315 observed, a decrease of 54.35% or 0.5 times lower than would be expected). It was not possible to perform similar analysis for Belgium or Germany due to legislation preventing collection of data on ethnicity or race in these countries. Although data on proportions of migrants and their country of origin is routinely collected, this does not account for naturalised children of foreign-born migrants.^14^ For this reason, population-level data were deemed insufficient to be usefully comparative.

## Discussion

### Under-reporting of ethnicity data in clinical vaccine trials

A key finding of our analysis was the under-reporting of ethnicity data in clinical vaccine trials registries across Europe. This may be associated with apprehension around collecting such data due to efforts to distance medical research from (particularly clear instances of) historical racism.^15 16^ Notable in our initial data collection was a higher rate of ethnicity reporting in the USA through the clinical trials registry compared with those of the UK or other European countries, or Europe collectively. US legislation on baseline characteristics for clinical trials—active encouragement or necessitating collection and reporting of ethnicity data—may also explain the disparity in reporting between the USA and Europe. Belgium diverged from this trend with a higher rate of ethnicity reporting despite being affected by the same misgivings as France and Germany regarding collection of such data.^17^ This may be accounted for by recent changes implemented in Belgium towards developing statistics on national origin including ascendancy.^18^ A lack of data hampers study into the extent of under-representation and is regularly acknowledged as an obstacle to addressing this issue.^19^ The lack of mechanisms to encourage reporting of participant demographics has also been noted. For example, ‘ClinicalTrials.gov’, the most comprehensive clinical trials database, does not require reporting of participant characteristics beyond age and sex^19^ - although a requirement to report race/ethnicity information *if collected* was introduced in April 2017.

### Under-representation of racial/ethnic minority groups in vaccine trials

The second notable finding was the under-representation of racial/ethnic minority groups in vaccine trials in Europe. All countries studied reported low percentages of ethnic minority participants, although the UK reported greater proportions compared with Belgium or Germany, for which under three percent of all participants were from ethnic/racial minority groups. Again, it is racism, especially exemplified through high profile historical events, which has been identified as a reason discouraging American ethnic minority groups from taking part in medical research.^20^ Similar factors may influence ethnic minority groups in Europe. It is also possible that disparity between the ethnic diversity of participants in trials conducted in Belgium, Germany and the UK is due to divergence in demographics of the wider populations of these countries.

Despite low representation of ethnic minority participants in clinical vaccine trials for Belgium and Germany, it is difficult to determine to what extent trial participants are representative of their wider populations, as there is no reliable data on the population-level ethnic demographics of either nation. Statistical analysis for the UK indicated that vaccine trial participants are not representative of the wider population, with particular under-representation of Asian and black or African American minority groups. This is consistent with research on the participation of ethnic minority groups in other forms of British medical research, and may diminish at a theoretical level the principles of justice and equity embedded in national guidelines on medical research.^21 22^ Additionally, this situation may have practical implications for the safety and effectiveness of routine vaccinations and thus pre-established health disparities within the UK population.

Evident is a greater recognition of the under-representation of ethnic minority groups in US clinical trials compared with other nations, with a clear majority of the literature focusing on American populations. Although this could be biased by the relative volume of research conducted in the USA, Europe does collectively produce more scientific literature (28.1% of global publications compared with 19.3% for the USA).^23^ Furthermore, an increased likelihood of recording race or ethnicity data in clinical trials registered on US databases has been described, with one study finding an approximately 10-fold increase in reporting between trials conducted in the US (40.3%) compared with western Europe (4.0%).^24^

Importantly, recognition can be correlated with action. In 1998, the US Food and Drug Administration ruled that recipients of government funding are required to compile data on participant demographics, including race. This was followed by policies from the Agency on Healthcare Research and Quality and the Centre for Disease Control on inclusion of women and minority groups in the research these bodies sponsored.^3^ One highly cited law was the National Institute of Health (NIH) Revitalisation Act of 1993, the first legislation mandating the inclusion of minority groups in NIH-funded clinical trials.^25^ The success of such legislation has been debated given the modest shifts resulting, but nonetheless marks recognition of this issue and a concerted effort to address it.^26^ The UK has no such regulation on inclusive clinical research.^27^ In 2017, the National Institute for Health and Care Research (NIHR) commissioned the NIHR Innovations in Clinical Trial Design and Delivery for the Under-served (NIHR-INCLUDE).^28 29^ Although the introduction of guidance on inclusive research in the UK is a significant touchstone, impact is difficult to assess given its recency. Furthermore, guidance, while useful, is unlikely to be as impactful as explicit regulation.

### Reasons for under-representation of racial/ethnic minority groups in vaccine trials

Many clinical trials require a high-performance status and absence of any comorbid conditions from organ dysfunction to mental health conditions for reasons of safety and ethics, and to ensure reliability of results through minimising confounders.^30 31^ In one case, comorbidity resulted in exclusion of close to one-in-five potential volunteers.^32^ Given that many ethnic minority groups have a well-established higher risk of comorbidity or multimorbidity, exclusion on this basis has been identified as a barrier to diverse recruitment when combined with provider attitudes.^33^

The attitudes of research staff or trial recruiters may play a role in limiting inclusion, as individuals from ethnic minority groups are often not viewed as ‘ideal’ potential participants, and clinicians holding race or ethnicity-based biases may affect patterns of referral for trials.^34^ Language also represents a key obstacle to inclusion; for example, a study of randomised controlled trials in Britain reported that 64% of excluded patients were unable to communicate in English.^27^ Certain groups may also face disproportionate logistical obstacles including costs of childcare or transportation. Research in the USA noted that large medical centres where clinical trials tend to be conducted rarely align geographically with areas where minority ethnic groups reside.^35^

Systemic barriers to desire precede or frame research opportunities and include generalised mistrust of medical research, medical professionals or figures of authority. A meta-analysis of a range of global qualitative studies cited mistrust as a barrier for 26% of participants.^36^ Historical experiences are argued throughout the literature to underpin this mistrust, in particular the Tuskegee syphilis trial, forced sterilisation of Black-American women, and the case of Henrietta Lacks whose cells were used to create the first immortalised human cell line without her consent.^37^,^41^ In the USA, incidences of historical racism clearly inform decisions around participation in clinical research, and social networks are cited as discouraging participation due to historical racism.^38^ Despite the prevalence of such factors in US literature, it is unclear whether similar factors are influential in other nations due to a lack of research on the topic.

Contextual barriers relate to specific conditions of a trial or recruitment strategies. Cultural naivety among researchers is regularly highlighted, with some forms of research having unrecognised significance for particular minority groups. The need for cultural sensitivity in clinical research is exemplified by the Havasupai Native Americans. Past disregard for spiritual values has fostered tensions with researchers; for example, although not uncommon in medical research, the Havasupai consider genotyping highly controversial.^42^ Cultural sensitivity should extend beyond oral communication to encompass awareness of body language; for example, individuals from some minority groups may find it inappropriate to shake hands with a clinician.^43^ Under-representation of ethnic minority participants may reflect the relative homogeneity in the ethnicity of research scientists,^35^ and some have suggested ‘race-matching’ recruiters to increase participation although studies dispute this, arguing characteristics such as openness and honesty are more influential.^44^

Similar to US literature, a range of factors may account for the under-representation of Asian and black or African American participants in vaccine trials in the UK. Lack of English comprehension has been cited as a significant barrier for the British South Asian community,^45^ together with perceived cultural differences from the researcher.^45 46^ Lower uptake of health services has also been reported among British black and Asian populations, limiting their access to clinical trial referrals.^47^ Furthermore, higher levels of comorbidities or multimorbidities among these groups in the UK relative to the majority white population may result in exclusion from trial participation.^48^ There are also a number of potential barriers to participation in trials stemming from research and recruitment design.^3549^,^53^ This could be a fruitful area for intervention as barriers such as language, expense, or location may be relatively easily mitigated.

### Possible solutions to under-representation of racial/ethnic minority groups in vaccine trials

A distinction can be made between proposed solutions to the under-representation of ethnic minority groups in clinical trials from (1) within clinical trials and their recruitment methodology and (2) external forces such as legislative bodies or funding sources. One recurring example of internal change was increased flexibility in inclusion criteria.^29 54^ Some advocate for relaxation of exclusion criteria for those with comorbidities or multimorbidities to prevent debarment.^29^ This may be particularly relevant to older adults, another understudied population,^55^ and may support early recognition of links between risk factors for comorbidity and intervention efficacy.^19^ While including comorbid participants is a safety concern, especially during phase I trials, increased flexibility when moving through trial stages could be beneficial. Flexibility is a theme that appears throughout the literature: temporal flexibility, flexibility in technology used and adaptation to cultural sensitivities.^33 54 56^ Each iteration emphasises the need to move beyond the rigid boundaries of trial conditions, addressing those outside the archetypal (and unrepresentative) participant.

External solutions concern encouraging or obliging those leading medical research to change. One example in the USA is government funding contingent on trial participant diversity, although reach is limited as a minority of phase III trials rely on government funding.^57^ Another suggestion is recruitment quotas; however, this has debatable ethical implications as it is clearly undesirable to motivate the pressuring of ethnic minority groups to participate, and there may be practical difficulties with recruiting sufficiently diverse participants from an ethnically homogeneous local population. One proposition is legislation against exclusion criteria disproportionately impacting ethnic minority groups. For example, research on NIHR mental health studies found 64% had a blanket exclusion against those unable to communicate in English.^27^ Soft approaches outside of industry have also been suggested such as working with religious or community networks.^33 58^ Such strategies are relatively low investment and without biological implications that could impact the merit of trial outcomes.

### Study limitations

Limitations of this study include the aggregation of racial/ethnic stratifications into broad groups and the use of US-derived definitions for data collection from clinical trials registries. This was a particular concern for the ‘Asian’ category, a term applied differently in the UK and the USA, and within which the UK census records data on five ethnic groups: Indian, Pakistani, Bangladeshi, Chinese and ‘any other Asian background’. Each group has its own distinct culture, social characteristics, and risk factors even without accounting for intragroup heterogeneity. There are clear disparities between theorisations of this issue and empirical research. For example, the literature tends to use terms related to ethnicity and self-identification, such as ‘Black-Caribbean’, whereas ethnicity reporting in clinical trials predominantly uses broader categories such as ‘black’. More granular ethnic categorisation appropriate to the population is required to improve understanding and inform the development of effective solutions to under-representation.

A further limitation was the relatively small number of trials available for analysis after excluding those without ethnicity data reported in the registries, although this in itself highlights a key issue. It is possible that the trials reporting ethnicity data are a self-selecting subset for which the representation of different ethnicities among participants is more likely to have been considered during recruitment. Those failing to report ethnicity data may be even less representative of the wider population. In some cases, ethnicity data may have been collected and even reported in publications but not reported in the clinical trials registries; this data was not captured in our analysis. Nonetheless, the total number of participants included in the analysis remained high (over 5000 in the UK).

A major barrier to broadening the applicability of our findings was the lack of population-level ethnic demographic data for Belgium or Germany. This prevented formal statistical analysis in the manner of the UK data, and frustrated drawing reliable conclusions on representation in Europe more widely. This is not an issue unique to these countries: of the three other most common vaccine trial locations in Europe (France, Italy and Spain) none record either ethnic or racial population data, instead favouring criteria such as citizenship.^59^ Explanations range from the historical weaponisation of racial data in Europe to efforts to de-emphasise individual variation in favour of a national identity. Discrimination on grounds of ethnic origin is considered the most pervasive form of discrimination within the European Union (EU), yet many countries have little idea of its impact.^59^ Thus, beyond the bounds of this study, a change to this framework of data collection should be advocated for.

Findings of this study are most relevant to vaccines intended for roll-out in the UK or Europe, particularly in the case of universal vaccination policies such as during the COVID-19 pandemic. However, this may not hold for all vaccine candidates, for example those developed against diseases such as malaria or tuberculosis that are regionally endemic. In such cases, it could be argued that trial participants should instead be representative of the demographics of the target country and thus early phase trials should be conducted there, but this requires a level of development and infrastructure that may be unattainable in resource-limited settings. Concern has also been reported among these populations feeling they may be exploited as ‘guinea-pigs’ by offshore researchers conducting first-in-man trials.^60^

### Conclusions

In conclusion, our quantitative analysis of vaccine trials registries data generated three key findings. First, it has shown that a significant majority of clinical trials fail to either record or report the ethnicity of their participants. Further research should explore whether this omission stems from trial management or lack of mechanisms for registering such information. Second, due to the availability of ethnic/racial data for the UK population, we were able to identify that participants in UK vaccine trials are not representative of the ethnic demographics of the wider population. This may have practical implications for the effectiveness of routine vaccinations, and may threaten the principles of justice and equity embedded in national guidelines on medical research. Finally, it has highlighted the difficulties of conducting research on inequities stemming from ethnicity in vaccine trials or medical research more broadly due to the lack of comparator data on ethnic demographics for many European countries. Without such data, it is difficult to assess the extent of the issue or effectively confront it.

## Data Availability

Data are available on reasonable request.
